# Supplementary material for: Thermotogota diversity and distribution patterns revealed in Auka and JaichMaa ‘ja ‘ag hydrothermal vent fields in the Pescadero Basin, Gulf of California
Source: PeerJ. 2024 Aug 19;12:e17724. doi: 10.7717/peerj.17724 (PMC11340630; doi:10.7717/peerj.17724)
Supplement: Supplemental Information 2 — Specific code employed for processing the genetic sequences obtained from the samples. This includes any algorithms or computational methods used in the analysis. [file peerj-12-17724-s002.gz › jm_methods_2024.html]

Pescadero-Basin


# Pescadero-Basin

#### Peña et al.

#### 2023/12/01

## Background

This is the supplemental methods accompanying the 16S rRNA amplicon analysis of sediment microbial communities from *JaichMaa ’ja ’ag* and *Auka* vent fields of the Pescadero Basin. Briefly, sediment cores were collected with an ROV from different sites within these vent fields and sectioned into 1-3ccm horizons. DNA was extracted, and amplicons of the V4V5 region of the 16S rRNA were amplified with 515F / 926R primers.

## Generating ASVs - DADA2

Raw reads were downloaded and placed in a directory `data_subset`

### Cutadapt

Primers were trimmed in bash with cutadapt. Reads without primers were discarded with `-m 100` and reads less than 100nt after trimming were also discarded.

```
MY_FILES=$(ls $PWD/data_subset/*R1*gz)  ### EDIT THIS FOR YOUR SAMPLES!!
echo -e "Today we are analyzing:\n\n$MY_FILES"
echo ""

mkdir -p fastq_cutadapt  # to make the directory where cutadapt-processed files will go

for R1 in $MY_FILES; do 
R2=$(echo $R1 | sed 's/_R1_/_R2_/')
cutadapt -a ^GTGCCAGCMGCCGCGGTAA...AAACTYAAAKRAATTGRCGG -A ^CCGYCAATTYMTTTRAGTTT...TTACCGCGGCKGCTGGCAC --discard-untrimmed -m 100 -o fastq_cutadapt/$(basename $R1) -p fastq_cutadapt/$(basename $R2) $R1 $R2
done
```

#### Reading in data to R

The previous cutadapt step put the trimmed data into a new directory `fastq_cutadapt`, which was then read and used to generate prefixes for all samples. Note that this contains both *Auka* and *JaichMaa ’ja ’ag* samples for a combined analysis.

```
library(dada2)
library(ggplot2)
library(reshape2)
library(dplyr)

forward_reads <- list.files("fastq_cutadapt", pattern="R1_001", full.names = T)
reverse_reads <- list.files("fastq_cutadapt", pattern="R2_001", full.names = T)

# this is names of files where filtered forward reads will be saved to, they're not filtered yet
filtered_forward_reads <- gsub("_001.fastq","_filtered.fastq", forward_reads)
filtered_reverse_reads <- gsub("_001.fastq","_filtered.fastq", reverse_reads)

# set up sample names
samples <- gsub("^.*/","", gsub("_S[0-9]*_L.*$", "", filtered_forward_reads)) 
print(samples)
```

Quality score were inspected to guide trimming lengths

```
plotQualityProfile(forward_reads[1:min(2, length(forward_reads))]) + 
  geom_vline(xintercept = 240, col='black', lty=2) +
  geom_hline(yintercept = 30, col='grey60', lty=3)
plotQualityProfile(reverse_reads[1:min(2, length(forward_reads))]) + 
  geom_vline(xintercept = 200, col='red', lty=2) +
  geom_hline(yintercept = 30, col='grey60', lty=3)
```

#### Filter & error rates

Based on the previous results we chose to truncate reads at 230 and 200 nt for R1 and R2, respectively, with `trunclen=c(230,200)`.

```
filtered_out <- filterAndTrim(forward_reads, filtered_forward_reads,
                reverse_reads, filtered_reverse_reads, maxEE=c(2,2),multithread = 16,
                rm.phix=TRUE, minLen=150, truncLen=c(230,200))

cbind(filtered_out, perc_kept = filtered_out[,2]/filtered_out[,1])
```

#### Estimate error models & denoise (make ASVs)

Using the filtered reads generated in the last step, error rates were estimated from a subset of the existing data Sequences are then dereplicated which `dada()` then uses with the error model to define ASVs and discard erroneous variants.

```
err_forward_reads <- learnErrors(filtered_forward_reads, multithread=16)
err_reverse_reads <- learnErrors(filtered_reverse_reads, multithread=16)

derep_forward <- derepFastq(filtered_forward_reads, verbose=FALSE)
derep_reverse <- derepFastq(filtered_reverse_reads, verbose=FALSE)

# the sample names in these objects are initially the file names, this sets them to the sample names
names(derep_forward) <- sample_names
names(derep_reverse) <- sample_names

# actual DADA2 denoising step
dada_forward <- dada(derep_forward, err=err_forward_reads, pool="pseudo", multithread=16)
dada_reverse <- dada(derep_reverse, err=err_reverse_reads, pool="pseudo", multithread=16)
```

#### Merge and chimera cleaning

The denoised forward and reverse reads were then merged. Chimeric sequences were identified and remove with `removeBimeraDenovo()`.

```
merged_amplicons <- mergePairs(dada_forward, derep_forward, dada_reverse, derep_reverse, 
                               trimOverhang=TRUE, minOverlap=12)
seqtab <- makeSequenceTable(merged_amplicons)

print("Merged fraction:")
rowSums(seqtab) / rowSums(makeSequenceTable(dada_forward))

seqtab.nochim <- removeBimeraDenovo(seqtab, verbose = T, method = 'consensus')

print("Non-chimeric fraction:")
sum(seqtab.nochim)/sum(seqtab)
```

#### Summarize read retention by step

To assess whether our parameterization for DADA2 commands were appropriate, we summarized the number of reads at each step to ensure there was no unexpected drastic loss at any step warranting adjustment of parameters.

```
getN <- function(x) sum(getUniques(x))

summary_tab <- data.frame(row.names=samples, samp=samples, input=filtered_out[,1],
               filtered=filtered_out[,2], dada_f=sapply(dada_forward, getN),
               dada_r=sapply(dada_reverse, getN), merged=sapply(merged_amplicons, getN),
               nonchim=rowSums(seqtab.nochim),
               final_perc_reads_retained=round(rowSums(seqtab.nochim)/filtered_out[,1]*100, 1))

summary_tab
write.table(summary_tab, "read-count-tracking.tsv", quote=FALSE, sep="\t", col.names=NA)

ggplot(melt(summary_tab[,-ncol(summary_tab)], variable.name='Step', value.name='Reads'), aes(x = Step, y = Reads)) +
    #geom_bar(aes(fill = Step), stat = 'identity') + 
  geom_boxplot(outlier.shape = NA) + geom_jitter(aes(color=samp), height=0, width=0.2) + guides(fill='none') +
  theme_bw() + theme(axis.text.x = element_text(angle = 90, color='black', hjust=1, vjust=0.5), 
                     axis.line.x = element_line(), axis.line.y = element_line())
ggsave("read-count-tracking.pdf", width=8, height=6)

melt(summary_tab[,-ncol(summary_tab)], variable.name='Step', value.name='Reads') %>% group_by(samp) %>%
  mutate(Reads=Reads/Reads[Step == "input"]) %>% mutate(guess_type='sample') %>% 
  mutate(guess_type=replace(guess_type, grep("[Bb]lank|[Cc]ont[rol]*|[Nn]eg|DNAex|50cyc",samp), 'control')) %>%
  ggplot(aes(x = Step, y = Reads)) +
  geom_boxplot(aes(color = guess_type), outlier.shape = NA) + theme_bw() +
  geom_point(position=position_jitterdodge(jitter.height=0, jitter.width=0.2), aes(fill=guess_type), alpha=0.6, pch=21) +
    theme(axis.text.x = element_text(angle = 90, color='black', hjust=1, vjust=0.5), panel.background = element_blank(),
          axis.line.x = element_line(), axis.line.y = element_line())
ggsave("read-count-tracking_percent.pdf", width=8, height=6)
```

### Annotation

Taxonomy was assigned using the SILVA r138 database amended with a set of unpublished full-length 16S sequences from methane seep samples.

```
library(DECIPHER)

# load in the latest SILVA dataset, lives at this path on OCEAN
load("/export/data1/db/16S_tag_processing_db/SILVA_SSU_r138_2019_orphanlab_k8-210919.RData")  # SILVA + orphan seqs

## creating DNAStringSet object of our ASVs
dna <- DNAStringSet(getSequences(seqtab.nochim))

## and classify, threshold=40 means 40% confidence as some lineages would be unclassified (default 60%)
tax_info <- IdTaxa(test=dna, trainingSet=trainingSet, strand="top", threshold=40, processors = 8)
```

At this point, the raw data was saved. Note that this set does not match the supplemental tables associated in the manuscript as contaminant ASVs have not yet been identified and removed.

```
 # giving our seq headers more manageable names (ASV_1, ASV_2...)
asv_seqs <- colnames(seqtab.nochim)
asv_headers <- vector(dim(seqtab.nochim)[2], mode="character")

for (i in 1:dim(seqtab.nochim)[2]) {
  asv_headers[i] <- paste(">ASV", i, sep="_")
}

  # making and writing out a fasta of our final ASV seqs:
asv_fasta <- c(rbind(asv_headers, asv_seqs))
write(asv_fasta, "ASVs.fa")

  # count table:
asv_tab <- t(seqtab.nochim)
row.names(asv_tab) <- sub(">", "", asv_headers)
write.table(asv_tab, "ASVs_counts.tsv", sep="\t", quote=F, col.names=NA)

  # tax table:
  # creating table of taxonomy and setting any that are unclassified as "NA"
ranks <- c("domain", "phylum", "class", "order", "family", "genus", "species")
asv_tax <- lapply(tax_info, function(x) {
  taxa <- unlist(strsplit(x$taxon, ";"))[-1]  # drop "Root"
  taxa <- c(taxa, rep(taxa[length(taxa)], max(0,length(ranks)-length(taxa))))
  taxa <- taxa[1:length(ranks)]  # pesky euks
})
asv_tax <- matrix(unlist(asv_tax), ncol=length(ranks), byrow = T, dimnames = list(names(asv_tax), ranks))
colnames(asv_tax) <- ranks
rownames(asv_tax) <- gsub(pattern=">", replacement="", x=asv_headers)

write.table(asv_tax, "ASVs_taxonomy.tsv", sep = "\t", quote=F, col.names=NA)
```

## Analysis

### Pre-processing ASV data

```
library(dada2)
library(ggplot2)
library(reshape2)
library(dplyr)
library(vegan)
library(dendextend)
library(tidyr)
library(viridis)
library(Biostrings)
library(DECIPHER)
library(phyloseq)
```

Counts, taxonomy, and metadata were read in.

```
counts_tab <- read.csv("ASVs_counts.tsv",header=T, row.names=1,check.names=F, sep="\t")
relative_tab <- apply(counts_tab, 2, function(x) 100*(x/sum(x)))
tax_tab <- read.csv("ASVs_taxonomy.tsv", header=T, row.names=1, check.names=F, sep="\t")
tax_tab$ASV <- rownames(tax_tab)
asv_seqs <- readDNAStringSet("ASVs.fa")

metadata <- read.csv('Metadata_FK181031.tsv', sep="\t")

stock_colors <- c("#FF0000","#F6A300","#0068CC","#6600AA","#AC0088","#AA33FF","#00FFFF","#00CC00","#006611","#00AC99",
                 "#AC6844","#FFFF00","#991100","#ACAC11","#a0f0aa","#FF00FF","#FF8611","#B9F6F6","#001166","#AC9A00",
                 "#994141","#ff1169","#0AF622","#119924","#Ac3311","#004A9A","#AcAc99","turquoise","tomato","sienna1",
                 "rosybrown","peachpuff","olivedrab3","mistyrose1","mediumorchid","indianred2","#114914","#660011",
                 "ivory3","deeppink","#331111")

site_colors <- c("Diane's vent"='#006680ff',
                 "Matterhorn"='#5fbcd3ff',
                 "North of Z vent"='#5f8dd3ff',
                 "South of Z vent"='#b7c4c8ff',
                 "Abuelita"='#ffaaaaff',
                 "Tay Uja and Weey 'kual"='#aa0000ff',
                 "Juwak Yuum"='#008000ff')
```

ASV names were updated to be in the format of “Family” + \_\_ + “#” were # is the rank order of the ASV by abundance.

```
tax_tab <- t(apply(tax_tab, 1, function(x) replace(x, which(x == 'uncultured'), x[sort(which(x != 'uncultured'), decreasing = T)[2]])))
tax_tab <- data.frame(tax_tab) %>% group_by(family) %>% mutate(ASV=paste(family, 1:length(family), sep="__"))
tax_tab <- data.frame(tax_tab)
rownames(tax_tab) <- tax_tab$ASV
rownames(counts_tab) <- rownames(tax_tab)
names(asv_seqs) <- rownames(tax_tab)
```

#### Treating controls with decontam

We used decontam to identify contaminant sequences using the prevalence method. As we are unware of reports of ANME being present as contaminants in kits, we manually removed ANME ASVs from the list of predicted contaminants.

```
predicted_controls <- grepl("[Bb]lank|[Cc]ont[rol]*|[Nn]eg|DNAex|50cyc|KC", colnames(counts_tab))

predicted_controls_names <- setNames(predicted_controls, colnames(counts_tab))

if (any(predicted_controls)){
  contam_predict <- decontam::isContaminant(t(counts_tab), method='prevalence', neg=predicted_controls, threshold=0.1)
  contam_asvs <- rownames(contam_predict)[contam_predict$contaminant] 
  apply(tax_tab[contam_asvs,], 1, function(x) paste0(x, collapse=";"))

  contam_asvs_anme_ids <- grep("ANME", tax_tab[contam_asvs,"family"])  # in case decontam gets some false positives
  contam_asvs <- contam_asvs[-contam_asvs_anme_ids]
  counts_tab <- counts_tab[!(rownames(counts_tab) %in% contam_asvs),]
  tax_tab <- tax_tab[!(rownames(tax_tab) %in% contam_asvs),]
  
  p <- relative_tab %>% melt(varnames = c('ASV','Sample'), value.name = 'relabund') %>% 
    mutate(decontam=factor(setNames(contam_predict$contaminant, rownames(contam_predict))[ASV], levels=c('TRUE','FALSE'))) %>% 
    group_by(decontam, Sample) %>% summarise(relabund=sum(relabund)) %>% ungroup() %>%
    mutate(library_type=replace(rep("Real", length(Sample)), predicted_controls, "Control")) %>% 
  mutate(Sample = factor(Sample, levels=unique(Sample[order(decontam,relabund)]))) %>%
  ggplot(aes(x=Sample, y=relabund, fill=decontam)) + geom_col() + scale_fill_manual(values=c("TRUE"='#ca2a00', 'FALSE'='black')) +
    facet_grid(.~library_type, scales='free_x', space='free_x') + scale_y_continuous(expand=c(0,0)) +
  theme_classic() + theme(axis.text.x = element_text(angle=90, hjust=1, vjust=0.5), axis.ticks.x = element_blank())
  
  print(p)
}
```

These finalized tables were then exported to become the tabs of the supplemental data files

```
write.csv(tax_tab, "SD1_tax.csv")
write.csv(counts_tab, "SD1_counts.csv")
```

### Figure 3 - NMDS

For this and all subsequent analyses, samples with less than 1000 reads were dropped, along with controls since they had been dealt with by decontam. The data was max-normalized by sample to obtain relative abundance as a percent value.

```
counts_tab <- counts_tab[,colSums(counts_tab) >= 1000] #Keep only samples with at least 1000 reads
counts_tab <- counts_tab[,!grepl("[Bb]lank|[Cc]ont[rol]*|[Nn]eg|DNAex|50cyc|KC", colnames(counts_tab))]
rownames(metadata) <- metadata$sample.id
```

A MDS was calculated using the family-level abundances using vegan’s `metaMDS` function with Bray-Curtis distances.

Figure 3A

```
makeMDSbyLevel <- function(counts=counts_tab, tax=tax_tab, level='family', distance='bray', try=100, trymax=500, autotransform=F) {
  counts <- cbind(tax[rownames(counts),], counts)
  counts <- dcast(melt(counts, variable.name='sample'), sample~get(level), value.var = 'value', fun.aggregate = sum)
  rownames(counts) <- counts$sample
  
  mds <- metaMDS(counts[,2:ncol(counts)], distance = distance, try = try, trymax = trymax, autotransform = autotransform)
  return(mds)
}

# generate MDS
mds <- makeMDSbyLevel(counts=counts_tab, level='family',try=100, trymax=200, distance='bray')
```

```
## Using domain, phylum, class, order, family, genus, species, ASV as id variables
```

```
## Run 0 stress 0.1989893 
## Run 1 stress 0.2049188 
## Run 2 stress 0.2006645 
## Run 3 stress 0.2029402 
## Run 4 stress 0.2008433 
## Run 5 stress 0.2018195 
## Run 6 stress 0.2019434 
## Run 7 stress 0.1995276 
## Run 8 stress 0.2022345 
## Run 9 stress 0.2009581 
## Run 10 stress 0.2004432 
## Run 11 stress 0.2006937 
## Run 12 stress 0.2009574 
## Run 13 stress 0.2029302 
## Run 14 stress 0.2042077 
## Run 15 stress 0.2005593 
## Run 16 stress 0.1984483 
## ... New best solution
## ... Procrustes: rmse 0.01544397  max resid 0.227454 
## Run 17 stress 0.2004313 
## Run 18 stress 0.2013394 
## Run 19 stress 0.2073226 
## Run 20 stress 0.2076915 
## Run 21 stress 0.1997597 
## Run 22 stress 0.1989908 
## Run 23 stress 0.2000442 
## Run 24 stress 0.1994808 
## Run 25 stress 0.1987887 
## ... Procrustes: rmse 0.01426355  max resid 0.1151056 
## Run 26 stress 0.2027268 
## Run 27 stress 0.2023752 
## Run 28 stress 0.2018644 
## Run 29 stress 0.2017642 
## Run 30 stress 0.1985654 
## ... Procrustes: rmse 0.01410466  max resid 0.1368454 
## Run 31 stress 0.2009173 
## Run 32 stress 0.201094 
## Run 33 stress 0.2032126 
## Run 34 stress 0.1989556 
## Run 35 stress 0.2000467 
## Run 36 stress 0.2019064 
## Run 37 stress 0.1986679 
## ... Procrustes: rmse 0.01005232  max resid 0.1396502 
## Run 38 stress 0.2019194 
## Run 39 stress 0.2005684 
## Run 40 stress 0.1981624 
## ... New best solution
## ... Procrustes: rmse 0.01249494  max resid 0.1388851 
## Run 41 stress 0.2001308 
## Run 42 stress 0.2026793 
## Run 43 stress 0.2012614 
## Run 44 stress 0.2059436 
## Run 45 stress 0.2017011 
## Run 46 stress 0.2020831 
## Run 47 stress 0.2014273 
## Run 48 stress 0.1989906 
## Run 49 stress 0.1990648 
## Run 50 stress 0.1988288 
## Run 51 stress 0.2004408 
## Run 52 stress 0.2009432 
## Run 53 stress 0.2005677 
## Run 54 stress 0.2008663 
## Run 55 stress 0.2007885 
## Run 56 stress 0.2013336 
## Run 57 stress 0.2134082 
## Run 58 stress 0.2041282 
## Run 59 stress 0.2013615 
## Run 60 stress 0.1996978 
## Run 61 stress 0.2061355 
## Run 62 stress 0.2006286 
## Run 63 stress 0.1995337 
## Run 64 stress 0.2025637 
## Run 65 stress 0.2016415 
## Run 66 stress 0.2041523 
## Run 67 stress 0.2019787 
## Run 68 stress 0.1996123 
## Run 69 stress 0.201221 
## Run 70 stress 0.2018925 
## Run 71 stress 0.2008111 
## Run 72 stress 0.2110526 
## Run 73 stress 0.2048543 
## Run 74 stress 0.199602 
## Run 75 stress 0.1997854 
## Run 76 stress 0.2030381 
## Run 77 stress 0.2026248 
## Run 78 stress 0.199314 
## Run 79 stress 0.1997147 
## Run 80 stress 0.2033159 
## Run 81 stress 0.2007518 
## Run 82 stress 0.2001722 
## Run 83 stress 0.2013985 
## Run 84 stress 0.1980088 
## ... New best solution
## ... Procrustes: rmse 0.009927716  max resid 0.1403055 
## Run 85 stress 0.2028356 
## Run 86 stress 0.2004376 
## Run 87 stress 0.1996915 
## Run 88 stress 0.1984768 
## ... Procrustes: rmse 0.006824817  max resid 0.1005806 
## Run 89 stress 0.1980102 
## ... Procrustes: rmse 0.001503872  max resid 0.0133541 
## Run 90 stress 0.20054 
## Run 91 stress 0.205091 
## Run 92 stress 0.2013677 
## Run 93 stress 0.199554 
## Run 94 stress 0.2052776 
## Run 95 stress 0.2009043 
## Run 96 stress 0.1996063 
## Run 97 stress 0.2026072 
## Run 98 stress 0.2624586 
## Run 99 stress 0.2013674 
## Run 100 stress 0.2011931 
## Run 101 stress 0.1984921 
## ... Procrustes: rmse 0.01007816  max resid 0.1066633 
## Run 102 stress 0.1997877 
## Run 103 stress 0.1987733 
## Run 104 stress 0.1987677 
## Run 105 stress 0.2008286 
## Run 106 stress 0.2007149 
## Run 107 stress 0.2027526 
## Run 108 stress 0.2006588 
## Run 109 stress 0.2010951 
## Run 110 stress 0.2010759 
## Run 111 stress 0.2018628 
## Run 112 stress 0.2006787 
## Run 113 stress 0.1998447 
## Run 114 stress 0.2024741 
## Run 115 stress 0.2645904 
## Run 116 stress 0.1993728 
## Run 117 stress 0.201114 
## Run 118 stress 0.1997268 
## Run 119 stress 0.1999357 
## Run 120 stress 0.2021221 
## Run 121 stress 0.2064658 
## Run 122 stress 0.1992064 
## Run 123 stress 0.210563 
## Run 124 stress 0.2011518 
## Run 125 stress 0.2029058 
## Run 126 stress 0.1981802 
## ... Procrustes: rmse 0.005098842  max resid 0.04982322 
## Run 127 stress 0.2009682 
## Run 128 stress 0.2004546 
## Run 129 stress 0.2011512 
## Run 130 stress 0.1983253 
## ... Procrustes: rmse 0.006696161  max resid 0.0688866 
## Run 131 stress 0.2022429 
## Run 132 stress 0.2060609 
## Run 133 stress 0.2000818 
## Run 134 stress 0.2027773 
## Run 135 stress 0.2003474 
## Run 136 stress 0.2045426 
## Run 137 stress 0.199096 
## Run 138 stress 0.2027719 
## Run 139 stress 0.2000829 
## Run 140 stress 0.2002757 
## Run 141 stress 0.1995253 
## Run 142 stress 0.2007644 
## Run 143 stress 0.2017193 
## Run 144 stress 0.2009988 
## Run 145 stress 0.1980101 
## ... Procrustes: rmse 0.001473407  max resid 0.01277495 
## Run 146 stress 0.201077 
## Run 147 stress 0.1994371 
## Run 148 stress 0.2007791 
## Run 149 stress 0.2020861 
## Run 150 stress 0.1992312 
## Run 151 stress 0.1997226 
## Run 152 stress 0.1985042 
## ... Procrustes: rmse 0.01556434  max resid 0.2289363 
## Run 153 stress 0.2027007 
## Run 154 stress 0.1995351 
## Run 155 stress 0.199497 
## Run 156 stress 0.1986679 
## Run 157 stress 0.2023443 
## Run 158 stress 0.205277 
## Run 159 stress 0.2061975 
## Run 160 stress 0.2001549 
## Run 161 stress 0.2007636 
## Run 162 stress 0.1980128 
## ... Procrustes: rmse 0.00169224  max resid 0.01782226 
## Run 163 stress 0.2003684 
## Run 164 stress 0.2015069 
## Run 165 stress 0.2005717 
## Run 166 stress 0.2007362 
## Run 167 stress 0.1992655 
## Run 168 stress 0.200759 
## Run 169 stress 0.1998577 
## Run 170 stress 0.2005109 
## Run 171 stress 0.1997179 
## Run 172 stress 0.2009723 
## Run 173 stress 0.2004998 
## Run 174 stress 0.2006073 
## Run 175 stress 0.1989468 
## Run 176 stress 0.2088677 
## Run 177 stress 0.2093576 
## Run 178 stress 0.2014094 
## Run 179 stress 0.2013067 
## Run 180 stress 0.198667 
## Run 181 stress 0.1995043 
## Run 182 stress 0.2048304 
## Run 183 stress 0.1986999 
## Run 184 stress 0.2024335 
## Run 185 stress 0.2066053 
## Run 186 stress 0.2002404 
## Run 187 stress 0.2027328 
## Run 188 stress 0.200833 
## Run 189 stress 0.2003718 
## Run 190 stress 0.2003202 
## Run 191 stress 0.2002559 
## Run 192 stress 0.2012768 
## Run 193 stress 0.1992163 
## Run 194 stress 0.2036029 
## Run 195 stress 0.2015048 
## Run 196 stress 0.2042648 
## Run 197 stress 0.2017139 
## Run 198 stress 0.1984575 
## ... Procrustes: rmse 0.00793308  max resid 0.1149518 
## Run 199 stress 0.2011541 
## Run 200 stress 0.1995797 
## *** Best solution was not repeated -- monoMDS stopping criteria:
##    109: no. of iterations >= maxit
##     91: stress ratio > sratmax
```

```
# extract positions, merge metadata
mds_plot <- data.frame(mds$points)
mds_plot$sample <- rownames(mds_plot)

mds_plot <- merge(mds_plot, metadata, by.x='sample', by.y='sample.id', all.x=TRUE)
rownames(mds_plot) <- mds_plot$sample
mds_plot <- mds_plot[rownames(mds$points),]

### calculate confidence ellipses
conf_ell <- ordiellipse(mds, mds_plot$Location, kind = 'sd', conf=0.95, draw = 'none')  # find center

# helper function calculate ellipse points
veganCovEllipse<-function (cov, center = c(0, 0), scale = 1, npoints = 100) 
  {
    theta <- (0:npoints) * 2 * pi/npoints
    Circle <- cbind(cos(theta), sin(theta))
    t(center + scale * t(Circle %*% chol(cov)))
}

# make matrix with coordinates for ellipse - need to change the $type column to match the category
df_ell <- data.frame()
for(g in unique(mds_plot$Location)){
  df_ell <- rbind(df_ell, cbind(as.data.frame(with(mds_plot[mds_plot$Location==g,],
                  veganCovEllipse(conf_ell[[g]]$cov,conf_ell[[g]]$center,conf_ell[[g]]$scale))),
                  Location=g))
}
colnames(df_ell)[1:2] <- c('MDS1','MDS2')

ggplot(mds_plot, aes(x=MDS1, y=MDS2, color=Site)) + 
  geom_point(aes (shape=Location)) + 
  geom_path(data=df_ell, color='black', aes(linetype=Location)) +
  scale_color_manual(values=site_colors) +
  coord_equal() + theme_bw() + theme(axis.text = element_text(color = "black"),
                          strip.background = element_rect(color = NA, fill = "gray90"), panel.background = element_rect(color = NA, fill = "white")) + labs(color = 'Site')
```

```
#ggsave("nmds.pdf", width=7.5, height=4.5, units = "in")
```

And Figure 3B by plotting the same ordination but coloring by magnesium concentration:

```
ggplot(mds_plot, aes(x=MDS1, y=MDS2, color=Magnesium)) + 
  geom_point(aes (shape=Location)) + 
  geom_path(data=df_ell, color='black', aes(linetype=Location)) +
  scale_color_viridis() +
  coord_equal() + theme_bw() + theme(axis.text = element_text(color = "black"),
                          strip.background = element_rect(color = NA, fill = "gray90"), panel.background = element_rect(color = NA, fill = "white")) + labs(color = 'Site')
```

### Figure 4 - Correlation

To identify candidates for further investigation, we correlated phylum-level relative abundances against porewater magnesium. Hydrothermal vent fluid is typically depleted in magnesium, so samples with low magnesium concentration likely are exposed to higher proportions of vent fluid. First, we max-normalized the data to obtain relative abundance data as a percentage.

```
relative_tab <- apply(counts_tab, 2, function(x) 100*(x/sum(x)))

relative_long <- melt(relative_tab, varnames = c("ASV","sample"), value.name = "relabund")

relative_long <- cbind(relative_long, tax_tab[as.character(relative_long$ASV), 
                                              grep("ASV", colnames(tax_tab),invert=T, value=T)])
```

#### Phylum-level overview

Supplemental figure

```
sub_relative_long <- relative_long %>% group_by(sample,phylum) %>% summarise(relabund=sum(relabund)) %>%
  merge(metadata, by.x="sample",by.y="sample.id") %>% 
  ungroup() %>% group_by(phylum) %>% filter(sum(relabund > 2) > 5) %>%
  group_by(Core, Dive) %>% filter(length(unique (Serial_Number)) > 3) %>% 
  mutate(Sample=paste0(Horizon, " [", Serial_Number,"]")) %>% 
  mutate(Sample = factor(Sample, levels=rev(gtools::mixedsort(unique(.$Sample),decreasing = TRUE)))) %>% ungroup() %>%
  group_by( Sample, Location, Site, Dive, Core) %>% group_modify(~add_row(.x, Sample=.x$Sample, Location=.x$Location, Site=.x$Site, 
                                                    Dive=.x$Dive, Core=.x$Core, phylum="Other phyla", relabund=100-sum(.x$relabund))) %>%
  group_by(phylum) %>% mutate(meanrelabund = mean(relabund)) %>% ungroup() %>%
  mutate(phylum=factor(phylum, unique(phylum[order(meanrelabund, decreasing = F)]))) %>% mutate(phylum=relevel(phylum, 'Other phyla'))
  
phylum_colors <- setNames(stock_colors[1:length(unique(sub_relative_long$phylum))], unique(sub_relative_long$phylum))
phylum_colors['Other phyla'] <- 'gray40'

ggplot(sub_relative_long[order(sub_relative_long$phylum),], aes(x=Sample, y=relabund, fill=phylum)) +
  geom_col() + labs(x="Sample (cmbsf)", y="Relative\nAbundance (%)") +
  ggh4x::facet_nested(.~ Location + Site + Dive + Core, scales = 'free', space = 'free') + 
  scale_fill_manual(values = phylum_colors)+
    theme_classic() +  
  theme(axis.text.x = element_text(angle=90, hjust=1, vjust=0.5, size=5), panel.spacing=unit(1,'mm'), 
        axis.text = element_text(color = "black"), strip.background = element_rect(color = NA, fill = "gray90"), 
        panel.background = element_rect(color = 'black', fill = "white"), 
        strip.background.y = element_blank(), strip.text.y = element_blank())
```

```
#ggsave('SFig1.pdf', width=22, height=6)
```

We correlated phylum-level abundances vs magnesium concentration for all phyla meeting our operational abundance criterion (more than 0.5% abundant in more 75 samples). The correlation was computed over all samples with magnesium concentrations, i.e., samples lacking an associated magnesium concentration were omitted.

```
rownames(metadata) <- metadata$sample.id

sampleschem <- metadata$sample.id[!is.na(metadata$Magnesium)]

cor_df <- relative_long %>% group_by(sample,phylum) %>% summarise(relabund=sum(relabund)) %>% group_by(phylum) %>% filter(sum(relabund>0.5) > 75) %>% dcast(phylum~sample)
```

```
## `summarise()` has grouped output by 'sample'. You can override using the
## `.groups` argument.
## Using relabund as value column: use value.var to override.
```

```
rownames(cor_df) <- cor_df$phylum #Change taxonomic level #original

cor_values <- cor(t(cor_df[,sampleschem]), metadata[sampleschem,'Magnesium'])

cor_values <- as.data.frame(cor_values)
cor_values$ASV <- rownames(cor_values)
cor_values <- cor_values[!is.na(cor_values$V1),]
```

The top correlations were obtained and plotted, this became Supplemental Figure 2.

```
cor_to_plot <- cor_values[cor_values$V1<0,]
cor_to_plot <- head(cor_to_plot[order(cor_to_plot$V1),])

sub_relative_long <- relative_long[relative_long$phylum %in% cor_to_plot$ASV,] %>% group_by(sample,phylum) %>% summarise(relabund=sum(relabund)) %>%
  merge(metadata,by.x="sample",by.y="sample.id") %>% filter(!is.na(Magnesium)) #Change location

ggplot(sub_relative_long[grep("Auka|JaichMaa", sub_relative_long$Location),], aes(x=Magnesium, y = relabund, color=Ammonium)) + #Change Color 
  geom_point() + 
  scale_x_continuous(expand=c(0,0), limits=c(0,70)) +
  scale_y_continuous(expand = c(0,0), limits=c(0,33)) +
  facet_grid(Location~phylum, scales = 'free_x', space = 'free_x') + 
  scale_colour_gradient(low = "black", high = "red") +
  xlab('Magnesium [mM]') + ylab('Relative Abundance (%)') + labs(color = 'Ammonium [mM]') +
  theme_bw() + 
  theme(axis.text.x = element_text(hjust=1, vjust=0.5), panel.spacing=unit(1,'mm'), axis.text = element_text(color = "black"), 
        strip.background = element_rect(color = NA, fill = "gray90"), panel.background = element_rect(color = NA, fill = "white"))
```

```
#ggsave("sfig_correlation.pdf", width=7.5, height=4.5, units = "in")
```

And Thermotogota were focused on for Figure 4AB:

```
ggplot(sub_relative_long[grepl("Auka|JaichMaa", sub_relative_long$Location) & grepl("Thermotogota", sub_relative_long$phylum),], 
       aes(x=Magnesium, y = relabund, color=Ammonium)) + #Change Color 
  geom_point() + 
  scale_x_continuous(expand=c(0,0), limits=c(0,70)) +
  scale_y_continuous(expand = c(0,0), limits=c(0,33)) +
  facet_wrap(~Location, scales='free') + 
  scale_colour_gradient(low = "black", high = "red") +
  xlab('Magnesium [mM]') + ylab('Relative Abundance (%)') + labs(color = 'Ammonium [mM]') +
  theme_classic() + 
  theme(axis.text.x = element_text(hjust=1, vjust=0.5), panel.spacing=unit(1,'mm'), axis.text = element_text(color = "black"), 
        strip.background = element_rect(color = NA, fill = "gray90"), panel.background = element_rect(color = NA, fill = "white"))
```

```
#ggsave("correlation.pdf", width=7.5, height=4.5, units = "in")
```

### Figure 5 - Thermotogota

Based on the intriguing correlation between Thermotogota and magnesium (a proxy for vent fluid), we decided to further explore Thermotogota ASV-level distribution across the Pescadero basin and within each core.

For this analysis, we were particularly interested in compositional shifts within Thermotogota, e.g., were most of the Thermotogota at Abuelita the same ASV or family; does the dominant Thermotogota member change with depth below seafloor or vent fluid concentration, etc? Thus, we normalized each Thermotogota ASV such that 100% = the total Thermotogota reads in that sample.

First, we subset to focus on abundant and prevalent Thermotogota - operationally, these were defined as ASVs comprising over 20% of the Thermotogota at least one sample and being at least 1% of total Thermotogota in at least 12 samples. We also removed any cores having less than 6 horizons, as these typically reflected cores with non-standard horizon intervals, i.e., more than the typical 1-3cm interals. We also removed mat samples.

```
sub_relative_long <- relative_long[grep("Thermotog", relative_long$phylum),]
sub_relative_long <- merge(sub_relative_long, metadata, by.x='sample', by.y='sample.id', all.x=TRUE)

ThermoASV <- sub_relative_long %>% group_by(sample) %>% mutate(relabund=100*relabund/sum(relabund)) %>% group_by(ASV) %>%
  mutate(maxabund=max(relabund,na.rm=TRUE)) %>% ungroup() %>% filter(!grepl('unclassified',ASV)) %>%
  filter(!grepl('[Mm]at',Horizon)) %>%
  filter(maxabund > 20) %>% group_by(ASV) %>% filter(sum(relabund > 1, na.rm=T) > 12) %>% droplevels() %>% 
  filter(Location %in% c('Auka',"JaichMaa 'ja 'ag")) %>% # Ja'ag'")) %>%  #1
  group_by(Core, Dive) %>% filter(length(unique (Serial_Number)) > 6) %>% 
  mutate(Sample=paste0(Horizon, " [", Serial_Number,"]")) %>% 
  mutate(Sample = factor(Sample, levels=rev(gtools::mixedsort(unique(.$Sample),decreasing = TRUE))))
```

Figure 5B To estimate the similarity between ASVs, we compared the percentage of identical bases in each ASV. This also produced a dendrogram useful for organizing the figure.

```
focus_ASVs <- as.character(unique(ThermoASV$ASV))
asv_dist <- 100*(1- DistanceMatrix(AlignSeqs(asv_seqs[focus_ASVs]), includeTerminalGaps = F))
```

```
## Determining distance matrix based on shared 8-mers:
## ================================================================================
## 
## Time difference of 0 secs
## 
## Clustering into groups by similarity:
## ================================================================================
## 
## Time difference of 0.01 secs
## 
## Aligning Sequences:
## ================================================================================
## 
## Time difference of 0.11 secs
## 
## Iteration 1 of 2:
## 
## Determining distance matrix based on alignment:
## ================================================================================
## 
## Time difference of 0 secs
## 
## Reclustering into groups by similarity:
## ================================================================================
## 
## Time difference of 0.01 secs
## 
## Realigning Sequences:
## ================================================================================
## 
## Time difference of 0.07 secs
## 
## Iteration 2 of 2:
## 
## Determining distance matrix based on alignment:
## ================================================================================
## 
## Time difference of 0 secs
## 
## Reclustering into groups by similarity:
## ================================================================================
## 
## Time difference of 0.01 secs
## 
## Realigning Sequences:
## ================================================================================
## 
## Time difference of 0.01 secs
## 
## ================================================================================
## 
## Time difference of 0 secs
```

```
asv_dist_order <- hclust(vegdist(asv_dist))

asv_dist <- as.data.frame(melt(asv_dist, varnames=c("ASV1","ASV2"), value.name="percid"))

asv_dist$ASV1 <- factor(asv_dist$ASV1, levels=asv_dist_order$labels[asv_dist_order$order])
asv_dist$ASV2 <- factor(asv_dist$ASV2, levels=asv_dist_order$labels[asv_dist_order$order])

asv_dist <- asv_dist[order(asv_dist$ASV1, asv_dist$ASV2),]

ggtree::ggtree(asv_dist_order, ladderize = F, right = F) + 
  ggtree::xlim(c(-1.01*max(asv_dist_order$height),4*max(asv_dist_order$height))) + ggtree::geom_tiplab() +  ## comment off this line if alignment is OK
  ggplot(asv_dist, aes(x=ASV1, y=ASV2)) + 
  geom_tile(aes(fill=percid)) + coord_equal() +
  scale_y_discrete(expand=c(0,0)) + scale_x_discrete(expand=c(0,0)) +
  scale_fill_gradient(low='lightgoldenrodyellow', high='red', na.value='#4a605a', name="% ID") +
  theme_classic() + theme(axis.text.x = element_text(angle=90, vjust=0.5, hjust=1), axis.text = element_text(color = "black"), strip.background = element_rect(color = NA, fill = "gray90"), panel.background = element_rect(color = NA, fill = "white"))
```

```
#ggsave("5B_Heatmap.pdf", width=10, height=4.5, units = "in")
```

For Figure 5A ASV proportions were then plotted as a heatmap:

```
ThermoASV$ASV <- factor(ThermoASV$ASV, levels=asv_dist_order$labels[asv_dist_order$order])

ThermoASV %>% mutate(subplot="ASV") %>%
  ggplot(aes(x=Sample, y=ASV)) +
  geom_tile(aes(fill = relabund)) + labs(y="ASV", x="Sample (cmbsf)", fill="Relative\nAbundance (%)") +
  geom_col(data=ThermoASV %>% group_by(Sample, Location, Site, Dive, Core) %>% 
             summarise(Magnesium=mean(Magnesium)) %>% mutate(ASV="Magnesium", subplot="0Mg"),
           aes(y=Magnesium, group=Core), fill='black') +
  ggh4x::facet_nested(subplot~ Location + Site + Dive + Core, scales = 'free', space = 'free') + 
  ggh4x::facetted_pos_scales(y=list(subplot == "Mg" ~ scale_y_continuous(expand=c(0,0), n.breaks = 2))) + 
  ggh4x::force_panelsizes(rows=c(1,12)) +
  scale_fill_gradient(low='white', high='turquoise4',  na.value = 'white') + theme_classic() + 
  theme(axis.text.x = element_text(angle=90, hjust=1, vjust=0.5, size=5), panel.spacing=unit(1,'mm'), axis.text = element_text(color = "black"),
        strip.background = element_rect(color = NA, fill = "gray90"), panel.background = element_rect(color = 'black', fill = "white"), 
        strip.background.y = element_blank(), strip.text.y = element_blank())
```

```
#ggsave("Thermotog_ASV_5A.pdf", width=13, height=5, units = "in")
```

#### Thermotogota without renormalization

Supplemental Figure

```
ThermoASV$ASV <- factor(ThermoASV$ASV, levels=asv_dist_order$labels[asv_dist_order$order])

sub_relative_long[sub_relative_long$ASV %in% ThermoASV$ASV,] %>% mutate(subplot="ASV") %>%
  mutate(Sample=paste0(Horizon, " [", Serial_Number,"]")) %>% 
  mutate(Sample = factor(Sample, levels=rev(gtools::mixedsort(unique(.$Sample),decreasing = TRUE)))) %>%
  filter(Location != 'Juwak Yuum') %>% group_by(Dive, Core) %>% filter(length(unique(Serial_Number)) > 6) %>%
  ggplot(aes(x=Sample, y=relabund)) +
  geom_col(aes(fill = ASV)) + 
  labs(fill="ASV", x="Sample (cmbsf)", y="Relative Abundance (%)") +
  geom_col(data=ThermoASV %>% group_by(Sample, Location, Site, Dive, Core) %>% 
             summarise(Magnesium=mean(Magnesium)) %>% mutate(ASV="Magnesium", subplot="0Mg"),
           aes(y=Magnesium, group=Core), fill='black') +
  ggh4x::facet_nested(subplot~ Location + Site + Dive + Core, scales = 'free', space = 'free') + 
  ggh4x::facetted_pos_scales(y=list(subplot == "0Mg" ~ scale_y_continuous(expand=c(0,0), n.breaks = 2))) + 
  ggh4x::force_panelsizes(rows=c(1,12)) +
  scale_fill_manual(values=stock_colors) +
  theme_classic() + 
  theme(axis.text.x = element_text(angle=90, hjust=1, vjust=0.5, size=5), panel.spacing=unit(1,'mm'), axis.text = element_text(color = "black"),
        strip.background = element_rect(color = NA, fill = "gray90"), panel.background = element_rect(color = 'black', fill = "white"), 
        strip.background.y = element_blank(), strip.text.y = element_blank())
```

```
#ggsave("Thermotog_ASV_S.pdf", width=13, height=5, units = "in")
```

#### Thermotogota phylogenetic tree

Supplemental figure

```
library(ape)
library(ggtree)
library(phytools)
```

```
tphy <- read.tree("phylogeny/thermo_commbined.fa-MUSCLE.treefile")
tphy <- midpoint.root(tphy)

branches <- setNames(grepl("__[0-9]*$",tphy$tip.label), seq(1, length(tphy$tip.label)))
tree <- groupOTU(tphy, branches, group_name = 'type')
```

```
basefontsize=1.8
ggtree(tree, layout = 'rect', lwd=0.1*basefontsize) + xlim(c(0,0.85)) + geom_tiplab(aes(color=type), size=basefontsize) + geom_nodelab(size=0.8*basefontsize) + scale_color_manual(values = c('black', '#6a0600'), guide='none') + 
  geom_treescale(fontsize = basefontsize, linesize = basefontsize*0.5, offset = basefontsize*1)
```

```
#ggsave("SFig4 .pdf", width=8.5, height=11)
```
