# Supplementary figures and images for: Thermotogota diversity and distribution patterns revealed in Auka and JaichMaa ‘ja ‘ag hydrothermal vent fields in the Pescadero Basin, Gulf of California
Source: PeerJ. 2024 Aug 19;12:e17724. doi: 10.7717/peerj.17724 (PMC11340630; doi:10.7717/peerj.17724)

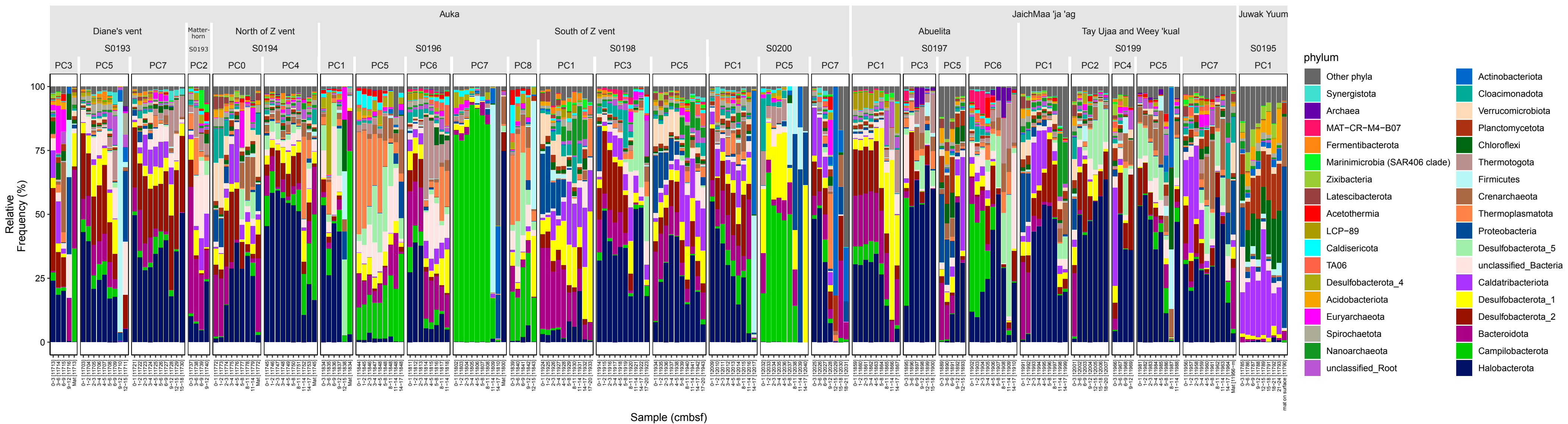

Supplement: Supplemental Information 6 — The relative frequencies of phyla derived from the 16S rRNA amplicon sequence variants found in the hydrothermal vent fields of Auka and JaichMaa ’ja ’ag. [file peerj-12-17724-s006.pdf]

# Auka

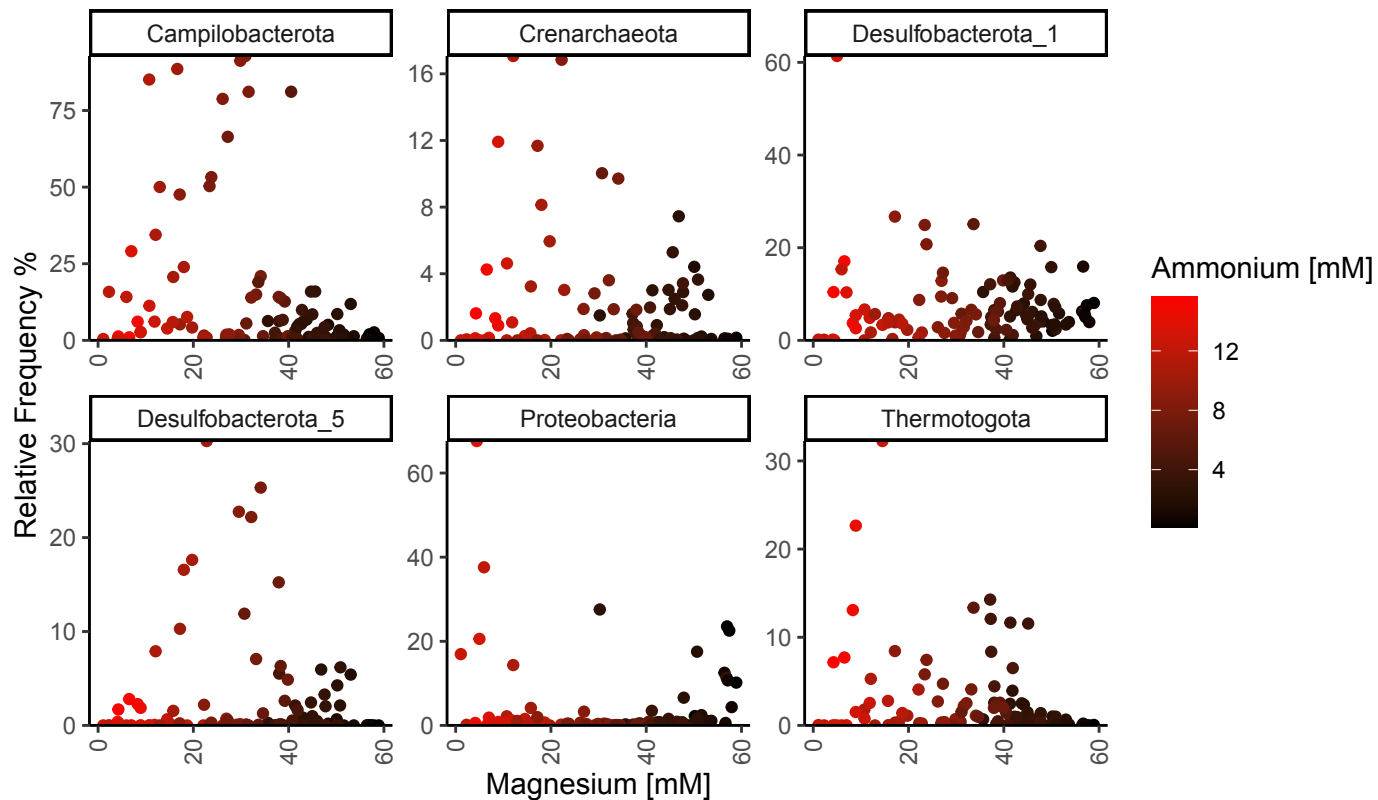

# JaichMaa Ja'ag'

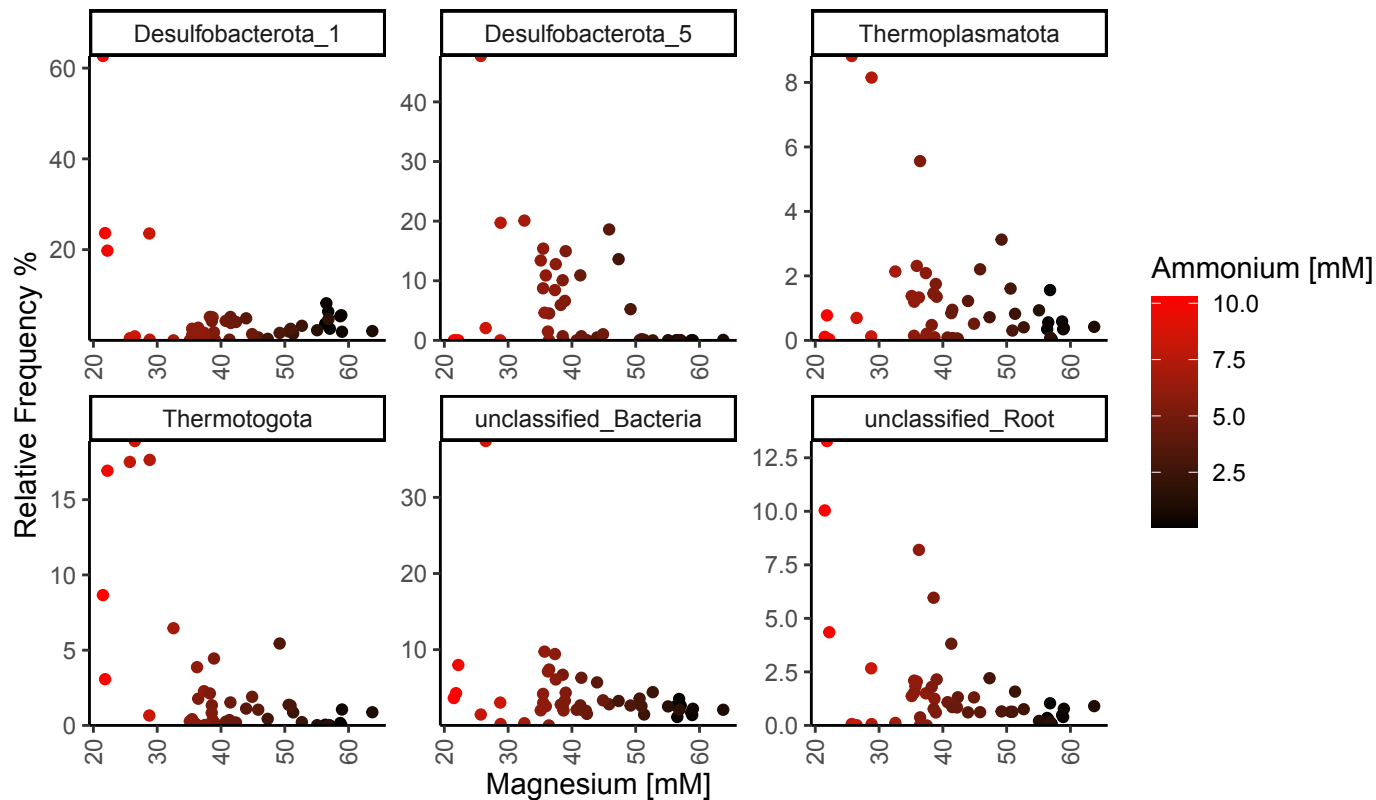

# Juwak Yuum

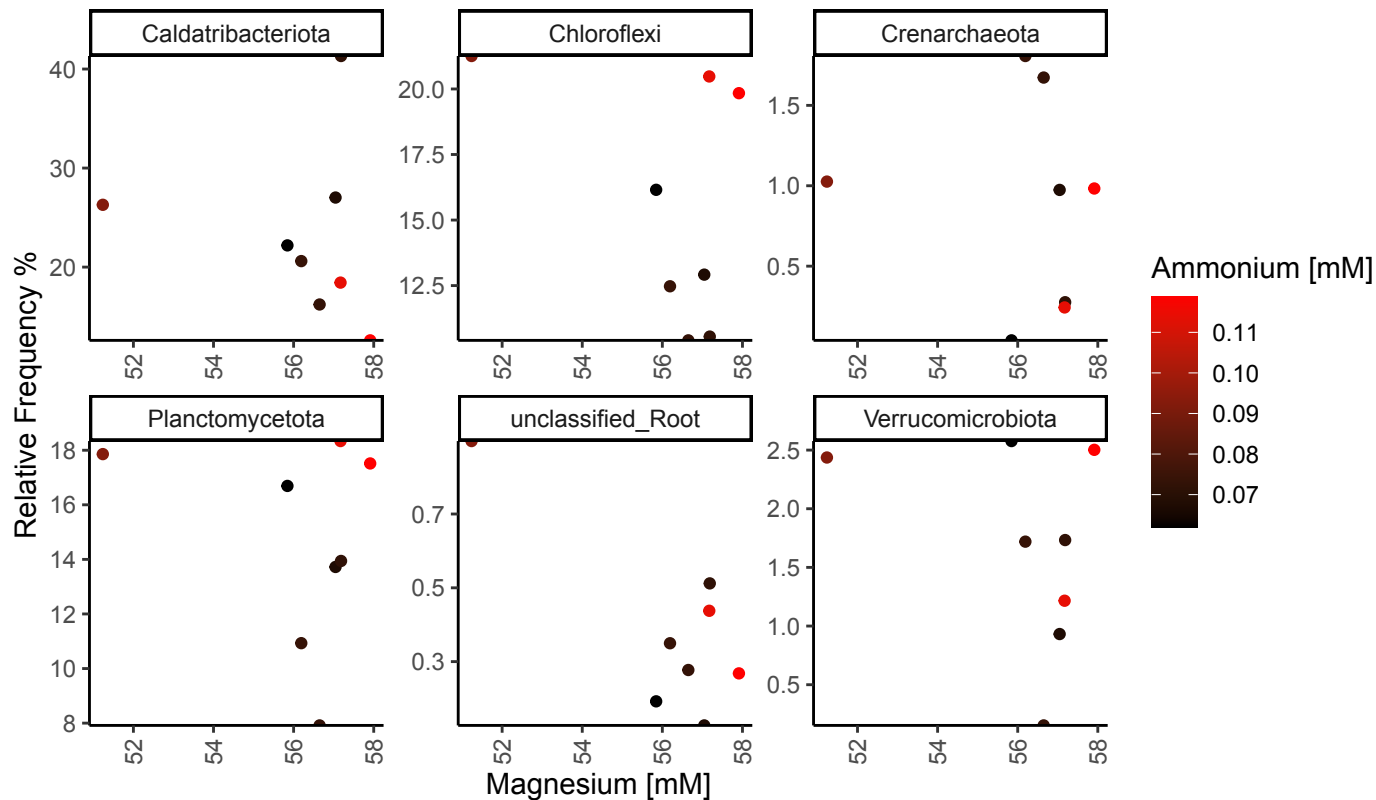

# Auka

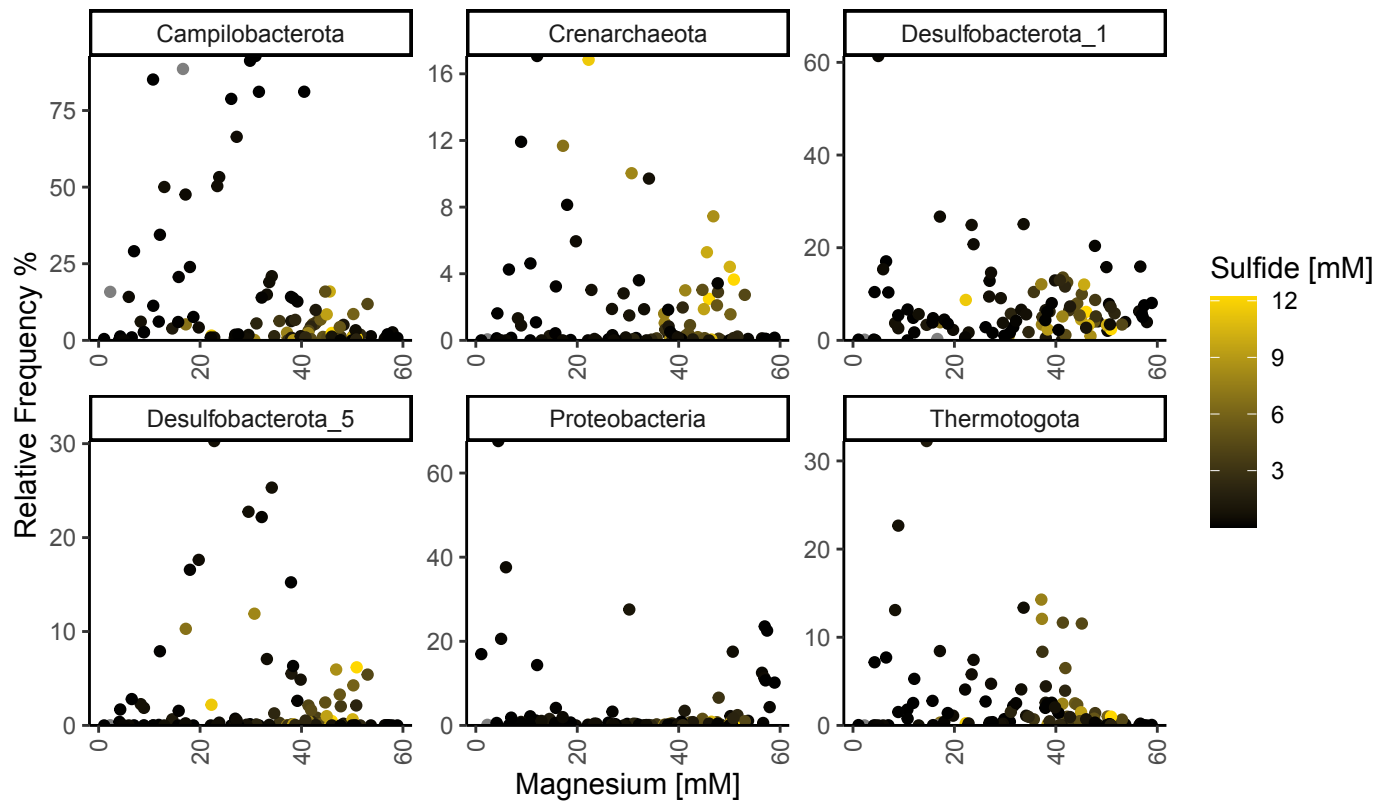

# JaichMaa Ja'ag'

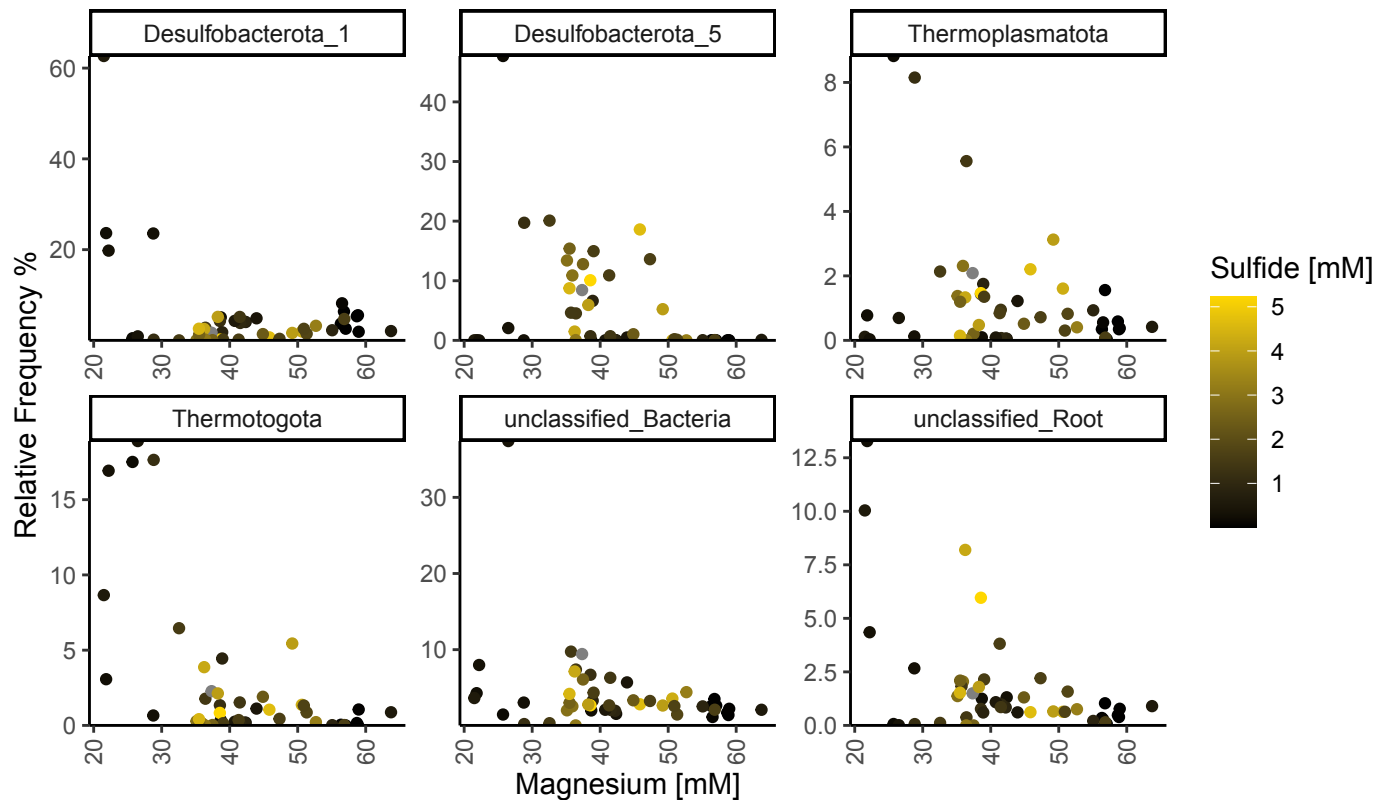

# Juwak Yuum

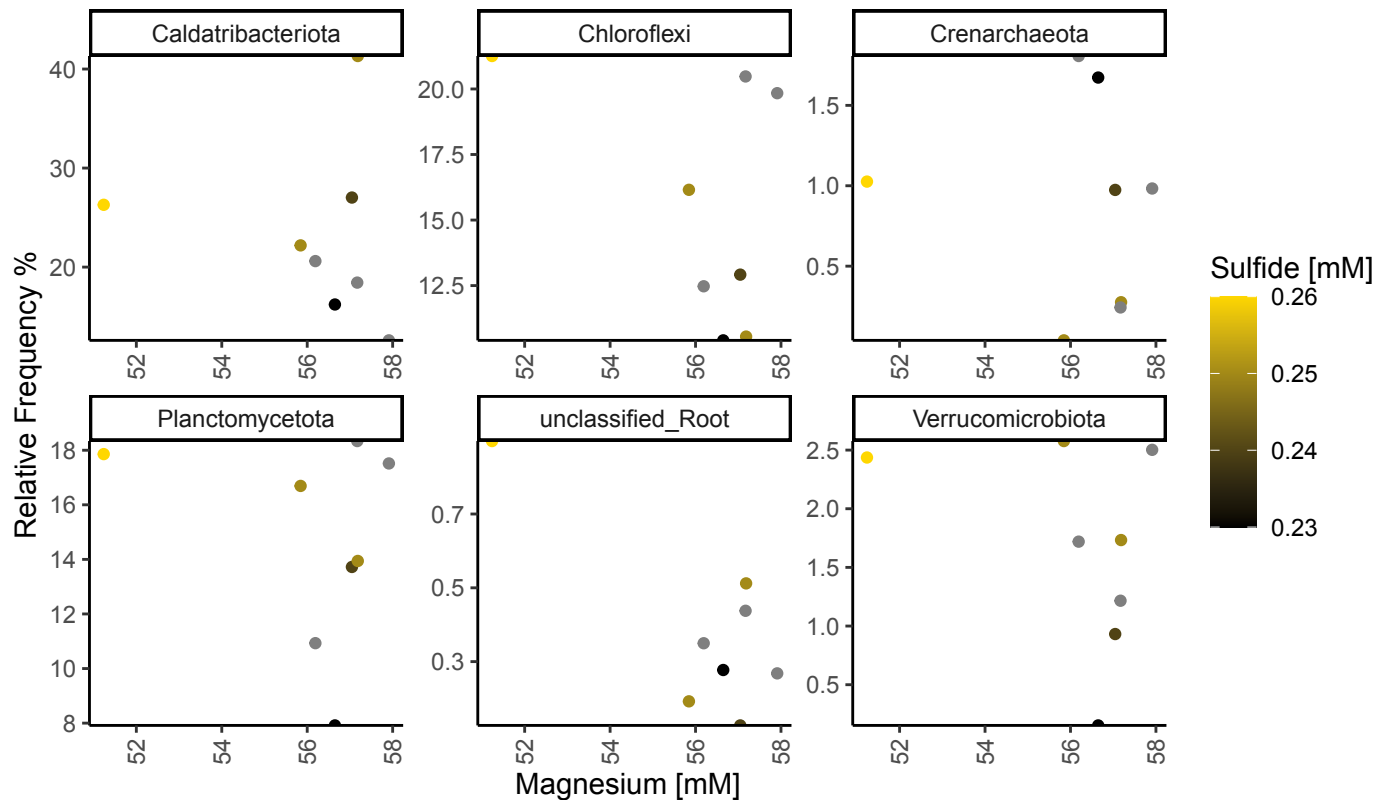

Supplement: Supplemental Information 7 — Microbial diversity correlated with hydrothermal fluids beneath the sediments in the Pescadero Basin hydrothermal vent fields. The most abundant Phyla with respect to magnesium [mM] in (A) Auka, (B) JaichMaa ‘ja ‘ag, and (C) Juwak Yuum with color coding based on ammonium and sulfide [mM]. All taxa correspond to Bacteria except for Crenarchaeota which corresponds to Archaea. The unclassified group could include both domains. [file peerj-12-17724-s007.pdf]

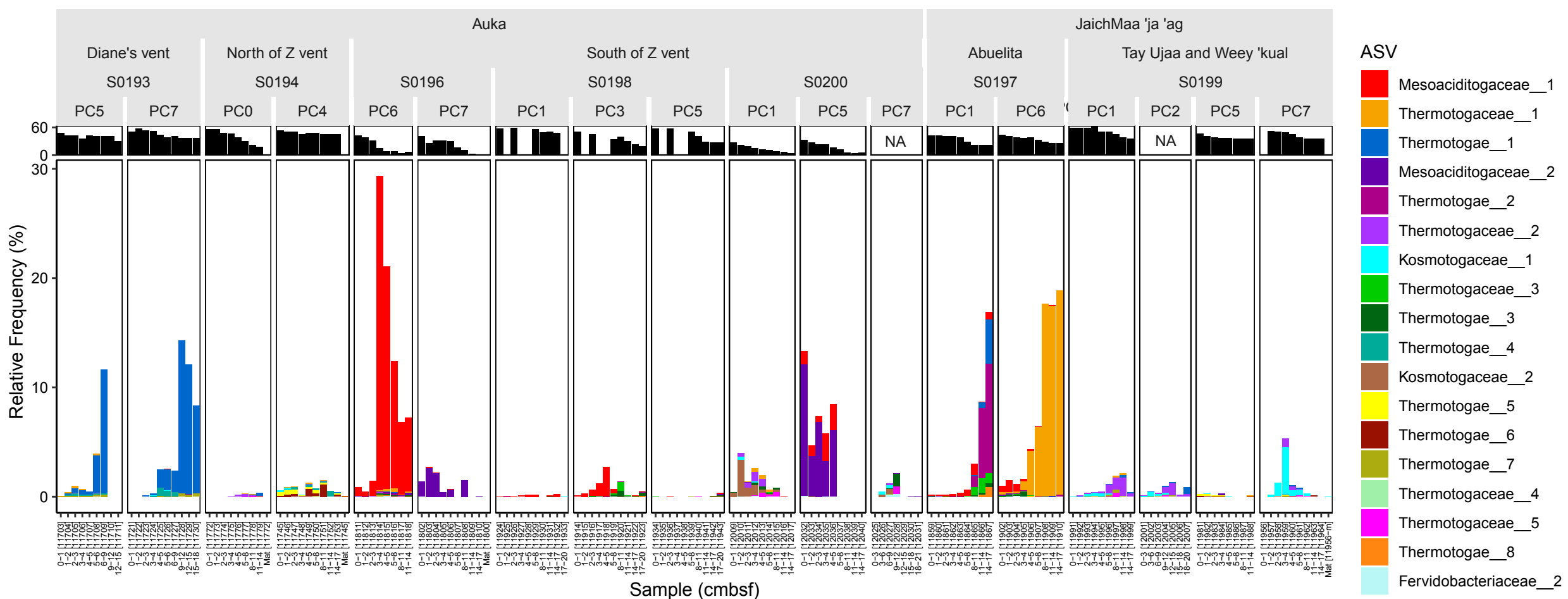

Supplement: Supplemental Information 8 — The relative frequency is no normalized to total Thermotogota abundance. [file peerj-12-17724-s008.pdf]

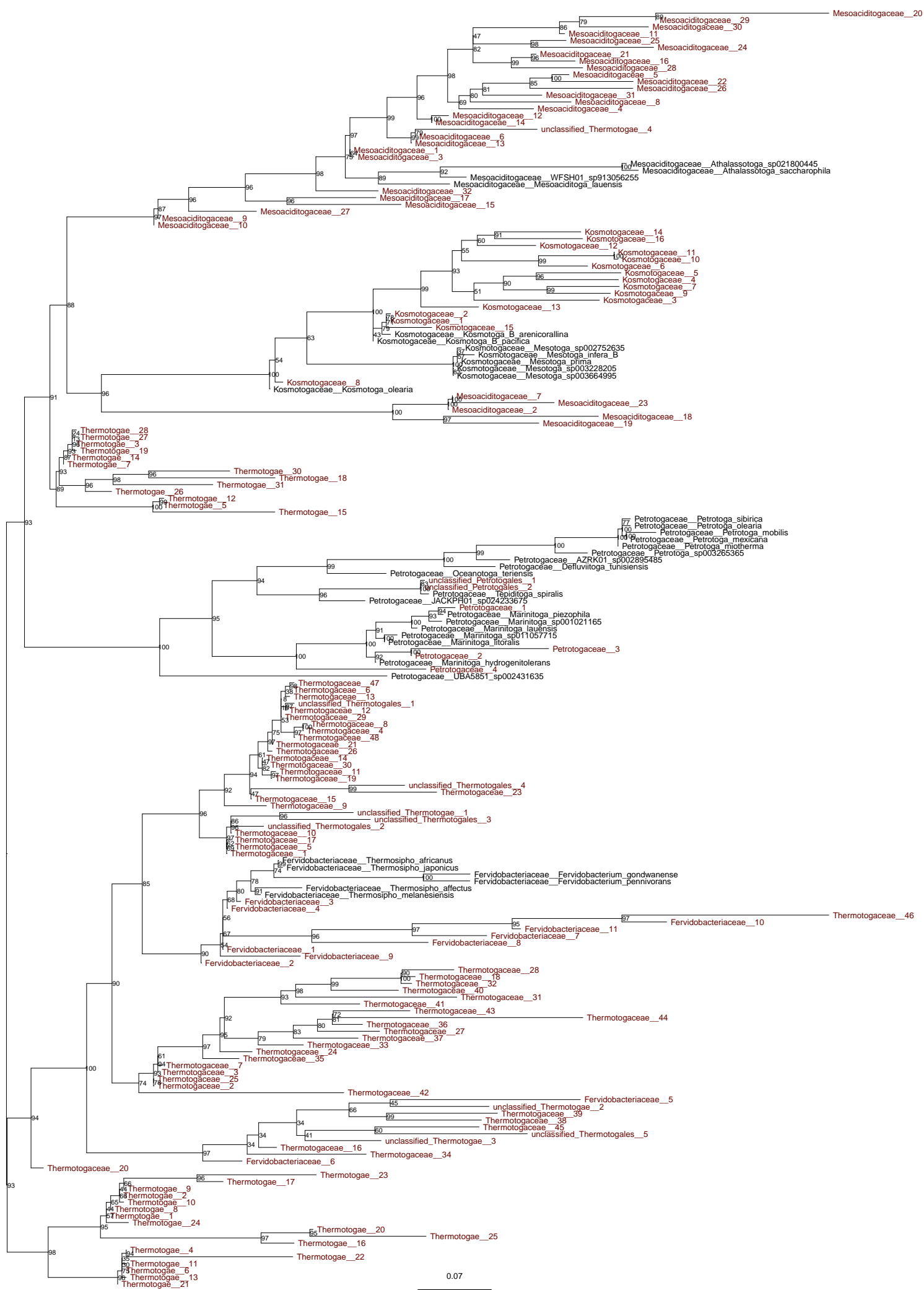

Supplement: Supplemental Information 9 — the tree illustrates the evolutionary relationships between Thermotogota ASV sequences obtained from the study (highlighted in red) and reference sequences. [file peerj-12-17724-s009.pdf]
